# Appropriate Rumen-Protected Glutamine Supplementation During Late Gestation in Ewes Promotes Lamb Growth and Improves Maternal and Neonatal Metabolic, Immune and Microbiota Functions

**DOI:** 10.3390/ani16010002

**Published:** 2025-12-19

**Authors:** Yifan Nie, Xiangjian Peng, Jiahao Li, Zhentiao Gao, Fei Zhang, Wei Jing, Yanfeng Liu, Cunxi Nie

**Affiliations:** 1 College of Animal Science and Technology, Shihezi University, Shihezi 832000, China; 15203934899@163.com (Y.N.); pxj1016pxj@sina.com (X.P.); xjauljh@163.com (J.L.); 15903033895@163.com (Z.G.); zhangfei_2023@163.com (F.Z.); banzhen299074@163.com (W.J.); 2 Institute of Feed Research, Xinjiang Academy of Animal Science, Urumqi 830011, China

**Keywords:** rumen-protected glutamine, fetal programming, lipid metabolism, amino acid metabolism, lambs

## Abstract

The gestational period is a critical developmental window where maternal nutrition programs offspring growth and long-term health. This study evaluated dietary rumen-protected glutamine (RP-Gln) supplementation during late gestation on lamb growth performance, and its regulatory effects on metabolic, immune, and microbial parameters in ewes and offspring. Compared with controls, moderate RP-Gln significantly improved lamb growth, increasing birth weight, 15-day body weight, and average daily gain (*p* < 0.01). In ewes, RP-Gln elevated serum GGT activity (*p* < 0.05), improved lipid metabolism, and reduced offspring muscular stress. Antioxidant capacity was enhanced in both ewes and lambs, with higher antioxidant enzyme activities and lower MDA levels (*p* < 0.05). RP-Gln also remodeled gut microbiota by promoting beneficial genera like *Lactobacillus* and *Faecalibacterium*, and reinforcing dam–offspring microbial transmission. Metabolomics revealed elevated polyunsaturated fatty acids, vitamin C, and tyrosine derivatives in ewes, and enhanced aromatic amino acid biosynthesis in lambs. In summary, glutamine supplementation in late-gestation ewes enhances maternal antioxidant and anti-inflammatory capacity, optimizes gut microbiota, and stimulates n-3 PUFA synthesis. These benefits are transmitted to the fetus, improving offspring antioxidant function, immune regulation, and intestinal health. Glutamine also promotes aromatic amino acid synthesis while inhibiting the tryptophan–kynurenine pathway, collectively supporting lamb growth and health.

## Simple Summary

During late gestation, optimal maternal nutrition plays a critical role in fetal development. This study demonstrated that supplementing pregnant ewes with an optimal dose of rumen-protected glutamine improved lamb birth weight and postnatal growth, enhanced antioxidant and immune function, and promoted a more beneficial healthier gut microbiome. These results offer sheep producers a viable feeding strategy to increase productivity and profitability. Researchers have also gained valuable insights into how functional amino acids can positively influence developmental programming in ruminants.

## 1. Introduction

The gestational period represents a critical developmental window that programmatically shapes offspring health, development, and production performance [1]. Maternal nutritional status during gestation exerts long-term metabolic reprogramming effects on offspring health [2]. In sheep, late gestation is characterized by rapid fetal growth, marked primarily by accelerated fat deposition and muscle development [3]. During this phase, fetal weight gain accounts for approximately 80% of total gestational growth, substantially increasing maternal metabolic burden and elevating nutritional demands for energy, protein, and essential micronutrients [4]. Inadequate nutrition during this period readily leads to restricted fetal development, reduced birth weight, and impaired survival of newborn lambs [5,6], ultimately limiting livestock production efficiency and economic returns.

Despite considerable advances in reproductive nutrition, a key scientific challenge remains: how to effectively align maternal metabolic homeostasis with offspring development through dietary interventions to achieve synergistic benefits. Beyond essential and non-essential amino acids, a class of “functional amino acids” plays vital roles in regulating key metabolic pathways [7]. Functional amino acids are those that, beyond serving as building blocks for proteins, play crucial roles in various biochemical processes that support health and physiological functions. Glutamine, a notable functional amino acid, is unique in containing two nitrogen atoms, conferring an exceptional capacity as a carbon and nitrogen donor [8]. This property underpins its involvement in diverse biochemical processes including protein synthesis [9], lipid metabolism [10], oxidative stress mitigation [11], inflammation regulation [12], and cellular energy provision [13]. Previous studies have demonstrated that supplementing a low-protein diet with 1% glutamine significantly enhanced the growth performance of weaned piglets, improved serum biochemical indices, strengthened antioxidant capacity, modulated plasma amino acid profiles, and positively influenced fecal microbiota composition [14]. Recent work further suggests that glutamine can alleviate amino acid imbalance and acid-base homeostasis disruption induced by low-protein diets, thereby improving growth in chickens [15]. Intra-amniotic administration of L-glutamine has also been shown to promote intestinal maturation and stimulate enteroendocrine activity in chicken embryos [16]. In ruminants, glutamine supplementation improved ruminal epithelial morphology and integrity, increased digestive enzyme activity, and enhanced fermentation and immune function in fattening lambs [17]. In weaned calves, low-dose glutamine supplementation resulted in greater average daily gain and higher glutathione peroxidase activity compared to high-dose treatment [18]. However, glutamine undergoes substantial degradation and utilization by ruminal microbiota [19], which prevents it from reaching the hindgut at the intended dosage and exerting its physiological functions. To circumvent metabolic interference from rumen microbes and enhance its bioavailability in the posterior intestinal tract, it is necessary to implement rumen-protection strategies to ensure its effective delivery. To date, the potential application of rumen-protected glutamine (RP-Gln) in pregnant ruminant nutrition remains underexplored, and its practical value and feasibility warrant systematic investigation. Furthermore, during late gestation, accelerated fetal development not only heightens maternal metabolic pressure but also induces oxidative stress [20], which increases the risk of maternal inflammation and immunosuppression [21]. These conditions may facilitate placental transfer of inflammatory cytokines [22], contributing to adverse outcomes such as postpartum hemorrhage, low birth weight, and fetal death [23].

Based on these findings, we hypothesize that RP-Gln supplementation will enhance maternal antioxidant capacity and optimize amino acid metabolism, thereby regulating immune status and remodeling the gut microbiota composition. We further anticipate that these maternal changes will be transmitted to offspring through mechanisms such as placental transfer and vertical microbiota transmission, leading to improved growth performance, with dose-dependent effects expected.

## 2. Materials and Methods

### 2.1. Experimental Design and Diets for Gestating Ewes

This study was conducted at the breeding base of the Gongnaisi Sheep Farm in Yining, Xinjiang, China (82°41′ E, 43°27′ N; altitude 800 m) between November 2024 and May 2025. The sheep used in this experiment were raised under a semi-grazing and semi-house-feeding management system, and the experiment was conducted during the house-feeding period. A total of 80 Chinese Merino fine-wool ewes (3rd–4th parity) with body weights ranging from 50 to 60 kg and body condition scores between 3.0 and 3.5 (on a 5-point scale) were selected. On day 90 of gestation, the ewes were randomly allocated to one of four dietary groups (n = 20 per group): a Control group (basal diet), a Low-dose group (Low; basal diet + 10 g/d/head of RP-Gln), a Medium-dose group (Mid; basal diet + 20 g/d/head of RP-Gln), and a High-dose group (High; basal diet + 30 g/d/head of RP-Gln). The RP-Gln supplement (supplied by Kangerquan Feed Co., Ltd., Hangzhou, China) contained ≥52% glutamine with a rumen bypass rate of ≥87.3%. The rumen bypass rate of RP-Gln was evaluated through an in vitro fermentation methodology, employing simulated rumen fluid under a 24 h incubation protocol. From gestation day 90 to 135, ewes received their respective experimental diets; thereafter, all animals were switched back to the basal diet until parturition. Ewes were fed three times daily (07:00, 13:00, and 19:00) with ad libitum access to water. All groups received an isoenergetic basal diet meeting 95% of the nutrient requirements for perinatal ewes to ensure consistent energy intake. This approach ensured that any observed effects of RP-Gln could be specifically attributed to alterations in metabolic homeostasis and energy partitioning efficiency, rather than to differences in energy intake. The basal diet (Table 1) was formulated to meet or exceed the nutrient and energy levels recommended in *Nutrition Requirements of Meat-type Sheep and Goat (NY/T 816-2021)* [24].

### 2.2. Sample Collection

Ten ewes per group were randomly selected for sample collection. Within two hours postpartum, blood samples (10 mL) were collected from the jugular vein of each ewe. At the same time, colostrum (15 mL) was obtained, and approximately 4 g of fecal samples was collected via rectal stimulation. On day 15 postpartum, mature milk was collected at 08:00 and 20:00, with 30 mL collected each time. Samples from the two time points were pooled at a 1:1 volume ratio. All milk samples (colostrum and pooled mature milk) were preserved with potassium dichromate and stored at 4 °C until analysis. Also, on day 15 after lambing, five lambs from each group were selected for blood sampling from the jugular vein and then euthanized. Rectal content was aseptically collected immediately after euthanasia. The heart, liver, kidneys, and spleen were exercised and weighed. The organ coefficient was calculated based on the pre-slaughter live weight as the ratio of organ weight to body weight. Blood samples from both ewes and lambs were placed in a light-protected incubator at 15 °C for 3 h. Blood samples were collected using standard serum collection tubes, and the serum was separated by centrifugation at 3000 rpm for 15 min. All serum and fecal samples were flash-frozen in liquid nitrogen and stored at −80 °C for subsequent analysis.

### 2.3. Milk Composition Analysis

The composition of colostrum and mature milk, including fat, protein, lactose, non-fat solids, and total solids, was analyzed using a milk composition analyzer (Lactoscan MCC, Milkotronic Ltd., Nova Zagora, Bulgaria).

### 2.4. Measurement of Serum Biochemical Parameters, Antioxidant Indicators, and Inflammatory Cytokines

Serum biochemical parameters were analyzed using an automatic biochemical analyzer (LABOSPECT 008 AS, Hitachi High-Tech Corporation, Tokyo, Japan). The measured parameters included alanine aminotransferase (ALT), total protein (TP), albumin (ALB), globulin (GLB), albumin-to-globulin ratio (A/G), aspartate aminotransferase (AST), γ-glutamyl transferase (GGT), triglycerides (TG), total cholesterol (TC), low-density lipoprotein (LDL), high-density lipoprotein (HDL), creatine kinase (CK), hydroxybutyrate dehydrogenase (HBDH), and lactate dehydrogenase (LDH). Serum antioxidant indicators—including malondialdehyde (MDA) content, superoxide dismutase (SOD) activity, glutathione peroxidase (GSH-Px) activity, and total antioxidant capacity (T-AOC)—were measured using commercial ELISA kits (Nanjing Jiancheng Bioengineering Institute, Nanjing, China) according to the manufacturer’s instructions. Similarly, serum inflammatory cytokine levels, comprising interleukin-4 (IL-4), IL-6, IL-10, tumor necrosis factor-α (TNF-α), and interferon-γ (IFN-γ), were determined using ELISA kits (Jiangsu Jingmei Biotechnology Co., Ltd., Yancheng, China) following the provided protocols. All absorbance readings for ELISA were performed with a full-wavelength microplate reader (Multiskan Sky, Thermo Fisher Scientific Inc., Waltham, MA, USA).

### 2.5. Microbial 16S rRNA Sequencing

Faecal samples collected from ewes and lambs were subjected to microbial genomic DNA extraction and subsequent 16S rRNA gene sequencing at Shanghai Majorbio Bio-Pharm Technology Co., Ltd. (Shanghai, China). The experimental procedures were performed following a previously described protocol with minor modifications [25]. In brief, genomic DNA was extracted from faecal samples, and the V3–V4 hypervariable region of the bacterial 16S rRNA gene was amplified using primers 338F (5′-ACTCCTACGGGAGGCAGCAG-3′) and 806R (5′-GGACTACHVGGGTWTCTAAT-3′) on an ABI GeneAmp^®^ 9700 PCR system. The resulting amplicons were purified, pooled in equimolar ratios, and sequenced on an Illumina MiSeq PE300 platform (Illumina, San Diego, CA, USA) in paired-end mode according to the manufacturer’s instructions.

### 2.6. Serum Metabolome Measurement

A 100 μL aliquot of each serum sample was mixed with 400 μL of ice-cold extraction solvent (acetonitrile: methanol = 1:1, *v*/*v*) containing 0.02 mg/mL L-2-chlorophenylalanine as an internal standard. After vortexing, the mixture was subjected to ice-bath sonication for 30 min, incubated at −20 °C for 30 min to precipitate proteins, and then centrifuged at 13,000× *g* for 15 min at 4 °C. The supernatant was collected and dried under nitrogen gas. The residue was reconstituted in 100 μL of a 1:1 (*v*/*v*) acetonitrile–water solution, followed by sonication for 5 min in an ice bath and centrifugation at 13,000× *g* for 10 min at 4 °C. The final supernatant was transferred for LC–MS/MS analysis. The LC–MS/MS analyses were performed using the UHPLC-Q Exactive HF-X system from Thermo Fisher Scientific.

### 2.7. Statistical Analysis

All data are presented as the mean ± standard error of the mean (SEM). Statistical analyses were performed using GraphPad Prism software (version 10.1; GraphPad Software Inc., San Diego, CA, USA). Differences between two groups were evaluated using Student’s *t*-test, and comparisons among multiple groups were conducted by one-way analysis of variance (ANOVA), followed by appropriate post hoc tests where applicable (Tukey’s HSD). Statistical significance was set at *p* < 0.05, with specific levels denoted as * *p* < 0.05, ** *p* < 0.01, and *** *p* < 0.001.

#### 2.7.1. Processing of Microbiome Data

Raw sequencing reads in FASTQ format were demultiplexed and quality-filtered using QIIME (v. 1.9.1). After removal of primer, barcode, and chimeric sequences with UCHIME (v. 4.2), high-quality sequences were aligned against the GreenGenes reference database. Operational taxonomic units (OTUs) were clustered at 97% similarity using UPARSE (v. 11). Alpha and beta diversity analyses were performed on the Majorbio Cloud Platform (www.majorbio.com). Alpha diversity within samples was evaluated using the Chao1 index (representing species richness) and the Shannon index (reflecting both richness and evenness). Beta diversity between samples was assessed based on Bray–Curtis distance and visualized via principal coordinate analysis (PCoA). Statistical differences in alpha and beta diversity among groups were determined using the Kruskal–Wallis H test. Linear Discriminant Analysis Effect Size (LEfSe) was applied to identify microbial taxa exhibiting differential abundance between groups, with screening thresholds set at *p* ≤ 0.05 (Kruskal–Wallis H test), LDA score > 2, and FDR < 0.05. The relative abundances of dominant bacterial taxa at the phylum and genus levels were also compared using the Kruskal–Wallis H test. All raw sequencing data have been deposited in the NCBI Sequence Read Archive under BioProject accession number PRJNA1294606.

#### 2.7.2. Processing of Metabolome Data

Raw LC–MS/MS data were processed using Progenesis QI software (v2.4; Waters Corporation, Milford, MA, USA) for peak detection, alignment, and normalization. Metabolites were identified by querying the HMDB and Metlin databases. The processed data matrix was imported into the Majorbio Cloud Platform (Majorbio, Shanghai, China) for subsequent analysis. Metabolic pathway enrichment and topology analyses were performed based on the KEGG database to interpret the biological roles of differentially abundant metabolites.

## 3. Results

### 3.1. Effects of RP-Gln Supplementation in Ewes During Late Gestation on Early Growth Performance and Organ Development of Offspring Lambs

**Figure 1:**
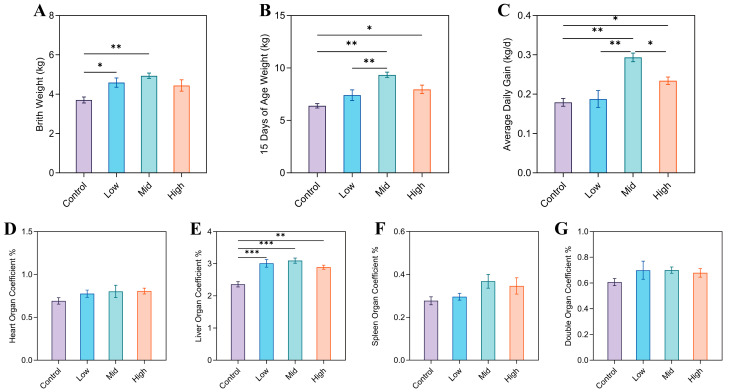
Growth performance and organ weight in lambs. (**A**) Birth weight. (**B**) Weight at 15 days of age. (**C**) Average daily gain. (**D**) Heart organ coefficient. (**E**) Liver organ coefficient. (**F**) Spleen organ coefficient. (**G**) Double kidney organ coefficient. * *p* < 0.05, ** *p* < 0.01, *** *p* < 0.001.

At birth, lamb body weight was significantly higher in the Mid group compared with the Control group (*p* < 0.01), and the Low group also exhibited a marked increase relative to the Control (*p* < 0.05; Figure 1A). By day 15, body weight in the Mid group surpassed that of both the Low and Control groups (*p* < 0.01), while the High group also showed a significant elevation over the Control (*p* < 0.05; Figure 1B). With respect to average daily gain (ADG), lambs in the Mid group demonstrated a significant improvement over all other groups (*p* < 0.05), and the High group also achieved higher ADG than the Control (*p* < 0.05; Figure 1C). Organ coefficient analysis revealed that liver index was significantly elevated in all RP-Gln supplementation groups compared with the basal diet group (*p* < 0.01). In contrast, no significant differences were detected in heart, kidney, or spleen indices among the groups (Figure 1D–G).

### 3.2. Effects of RP-Gln Supplementation in Ewes During Late Gestation on the Composition of Colostrum and Mature Milk

Comparisons of colostrum composition revealed that fat content was significantly higher in the Mid and Control groups than in the Low group (*p* < 0.01; Table 2). No significant differences were detected among groups for the other components measured in colostrum and mature milk, including protein, lactose, non-fat solids, and total solids (Table 2).

### 3.3. Effects of RP-Gln Supplementation in Ewes During Late Gestation on Serum Biochemical Parameters in Ewes and Their Offspring

In ewes, serum γ-glutamyl transferase (GGT) activity was significantly elevated in the Mid group compared with the Control group (*p* < 0.05). No significant differences were observed among groups for the other 12 biochemical parameters measured, including alanine aminotransferase (ALT), total protein (TP), albumin (ALB), aspartate aminotransferase (AST), and triglycerides (TG) (Table 3). In lambs, GGT activity was significantly higher in the Mid group than in all other groups (*p* < 0.05), whereas ALT activity was elevated in the Low group compared with both the Mid and High groups (*p* < 0.05; Table 3). With respect to lipid metabolism, total cholesterol (TC) and low-density lipoprotein (LDL) levels were significantly higher in the Mid and High groups than in the Control group (*p* < 0.01). High-density lipoprotein (HDL) was also increased in the High Group relative to the Control (*p* < 0.05; Table 3). For indicators of muscle energy metabolism, creatine kinase (CK) activity was significantly lower in the Mid and High groups than in the Control and Low groups (*p* < 0.001; Table 3).

### 3.4. Effects of RP-Gln Supplementation in Ewes During Late Gestation on Serum Antioxidant Indicators and Inflammatory Cytokines in Ewes and Their Offspring

**Figure 2:**
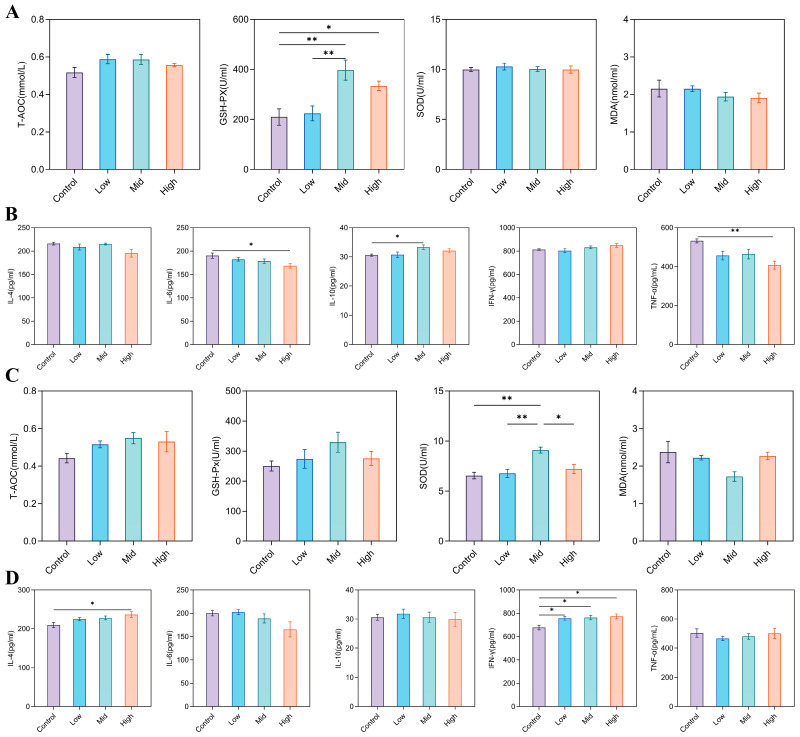
Antioxidant parameters and inflammatory cytokine levels in ewes and lambs. (**A**) Antioxidant parameters in ewes: T-AOC, GSH-Px, SOD, MDA. (**B**) Inflammatory cytokine levels in ewes: IL-4, IL-6, IL-10, IFN-γ, TNF-α. (**C**) Antioxidant parameters in lambs: T-AOC, GSH-Px, SOD, MDA. (**D**) Inflammatory cytokines in lambs: IL-4, IL-6, IL-10, IFN-γ, TNF-α. * *p* < 0.05, ** *p* < 0.01.

Maternal RP-Gln supplementation significantly enhanced systemic antioxidant capacity and modulated serum inflammatory cytokine profiles in both ewes and their offspring. In ewes, glutathione peroxidase (GSH-Px) activity was significantly increased in the Mid and High groups compared with the Control group (*p* < 0.05), while malondialdehyde (MDA) content was lowest in these two groups (Figure 2A). In addition, the pro-inflammatory cytokines IL-6 and TNF-α were significantly lower in the High group than in the Control (*p* < 0.05), whereas the anti-inflammatory cytokine IL-10 was markedly elevated in the Mid group (*p* < 0.05; Figure 2B). In lambs, superoxide dismutase (SOD) activity was significantly higher in the Mid group than in all other groups (*p* < 0.05). Both GSH-Px activity and total antioxidant capacity (T-AOC) peaked in the Mid group, which also exhibited the lowest MDA content among all groups (Figure 2C). Analysis of inflammatory cytokines showed that IL-4 was significantly elevated in the High group compared with the Control (*p* < 0.05), while IFN-γ levels in the Control group were significantly lower than those in all RP-Gln-supplemented groups (*p* < 0.05; Figure 2D).

### 3.5. Effects of RP-Gln Supplementation in Ewes During Late Gestation on the Rectal Fecal Microbiota of Ewes and Their Offspring

**Figure 3:**
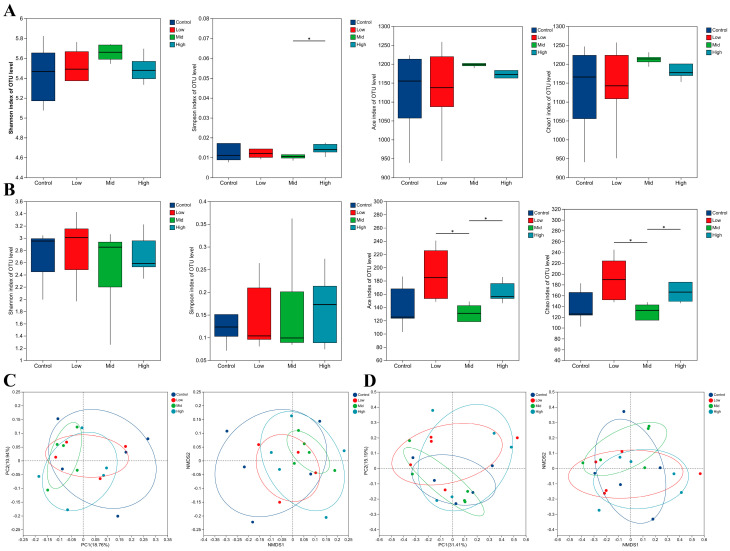
Analysis of alpha and beta diversity of the rectal fecal microbiota in ewes and lambs. (**A**) Alpha diversity indices in ewes: Shannon, Simpson, Ace, and Chao1. (**B**) Alpha diversity in lambs: Shannon, Simpson, Ace, and Chao1. (**C**) Beta-diversity analysis of the ewes’ rectal fecal microbiota was performed based on two-dimensional scatter plots of principal coordinate analysis (PCoA) and non-metric multidimensional scaling (NMDS) using Bray–Curtis distance matrices. (**D**) Beta-diversity analysis of the lambs’ rectal fecal microbiota was performed based on two-dimensional scatter plots of principal coordinate analysis (PCoA) and non-metric multidimensional scaling (NMDS) using Bray–Curtis distance matrices. * *p* < 0.05.

Alpha diversity analysis revealed no significant differences in Shannon, Ace, or Chao1 indices among the treatment groups in ewes (*p* < 0.05, Figure 3A). However, the Simpson index was significantly lower in the Mid group compared to the High group (*p* < 0.05), suggesting a reduction in community evenness at the medium dose level. In offspring lambs, no significant intergroup differences were observed in the Shannon or Simpson indices, whereas the Ace and Chao1 indices in the Mid group were significantly lower than those in the Low and High groups (*p* < 0.05, Figure 3B), indicating that medium-dose RP-Gln treatment led to a decrease in species richness. Beta diversity of the rectal faecal microbiota in ewes and lambs was assessed using principal coordinate analysis (PCoA) and non-metric multidimensional scaling (NMDS) based on Bray–Curtis distance matrices. In ewes, the first two principal coordinates (PC1 and PC2) in PCoA accounted for 18.76% and 10.94% of the variance, respectively, and NMDS revealed a similar distribution pattern. All treatment groups (control, low-, mid-, and high-dose) were clearly separated from the control group in the two-dimensional space, indicating a significant treatment effect with a non-linear dose–response relationship (Figure 3C). In lambs, PC1 and PC2 explained 31.41% and 15.19% of the variance, respectively, and NMDS yielded consistent results. A clear separation was observed between treatment groups and the control group, with the low- and mid-dose groups clustering closely, while the high-dose group occupied a distinct position, further supporting a non-linear dose effect (Figure 3D). The consistency between the PCoA and NMDS results reinforces the reliability of these findings.

### 3.6. Effects of RP-Gln Supplementation in Ewes During Late Gestation on Rectal Microbiota Composition and Core Microbiota in Ewes and Their Offspring

**Figure 4:**
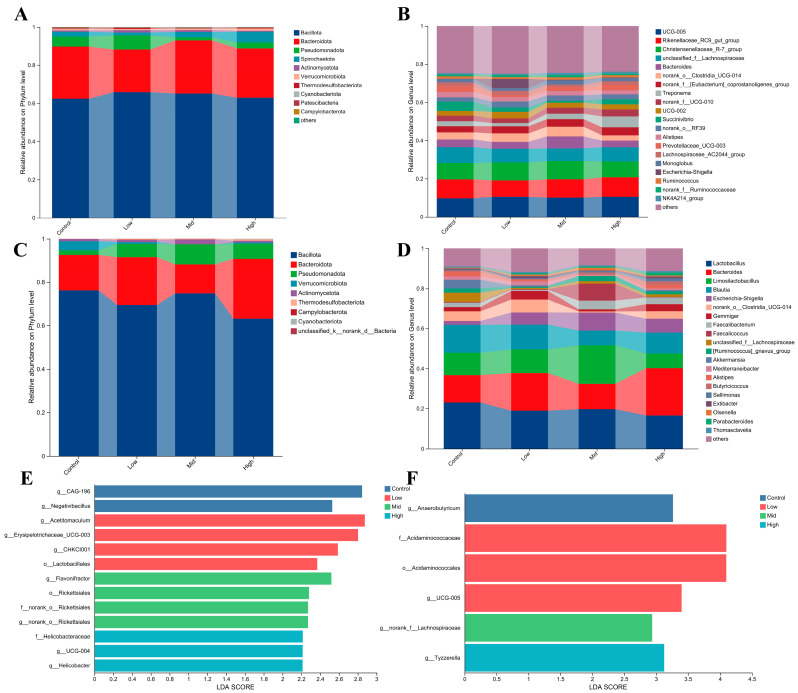
Composition of rectal fecal microbiota and LEfSe multilevel species discriminant analysis in ewes and lambs. (**A**) Composition of rectal fecal microbiota at the phylum level in ewes. (**B**) Composition of rectal fecal microbiota at the genus level in ewes. (**C**) Composition of rectal fecal microbiota at the phylum level in lambs. (**D**) Composition of rectal fecal microbiota at the genus level in lambs. (**E**) LDA discriminant bar plot for ewes. (**F**) LDA discriminant bar plot for lambs.

Microbial community composition was assessed via 16S rRNA high-throughput sequencing. In lambs, the rectal microbiota was predominantly composed of the phyla *Bacillota* and *Bacteroidota* (Figure 4C). Lambs in the Mid group exhibited a relative abundance of *Bacillota* reaching 74.9%, while *Bacteroidota* accounted for 13.3% (Figure 4C). RP-Gln supplementation was associated with a slight increasing trend in the phylum *Pseudomonadota* among lambs (Figure 4C), whereas in ewes of the Mid group, its abundance was significantly reduced to 1.6% (Figure 4A). At the genus level, the relative abundances of lactic acid bacteria—including *Lactobacillus* (19.6%), *Limosilactobacillus* (19.3%), and *Faecalibacterium* (4.3%)—were elevated in Mid group lambs compared with the Control, while *Blautia* and *Bacteroides* showed decreasing trends (Figure 4D). The rectal microbiota of ewes also responded to RP-Gln intervention: the Mid group displayed an 11.4% increase in *Christensenellaceae R-7* group, a modest rise in *Oscillospiraceae UCG-005*, and significant reductions in unclassified *Lachnospiraceae* and *Pseudomonadota* (Figure 4B). LEfSe analysis further identified signature microbial taxa across dosage groups. In ewes, the butyrate-producing genus *Flavonifractor* was significantly enriched in the Mid group, while the Control group was dominated by unclassified *Pseudomonadota* and *CAG-196* (Figure 4E). The Low group showed enrichment of *Lactobacillales*, *Acetitomaculum*, *CHKCI001*, and *Erysipelotrichaceae UCG-003*, whereas the High group exhibited elevated abundance of the potential pathogen *Helicobacter* and unclassified Oscillospirales (Figure 4E). In lambs, the Mid group was characterized by significant enrichment of several genera within *Lachnospiraceae*, the High group showed higher abundance of *Tyzzerella*, the Low group was enriched with the order *Acidaminococcales* and *Oscillospiraceae UCG-005*, and the Control group was primarily represented by *Anaerobutyricum* (Figure 4F).

### 3.7. Serum Metabolic Profiling in Ewes and Lambs Following RP-Gln Supplementation

**Figure 5:**
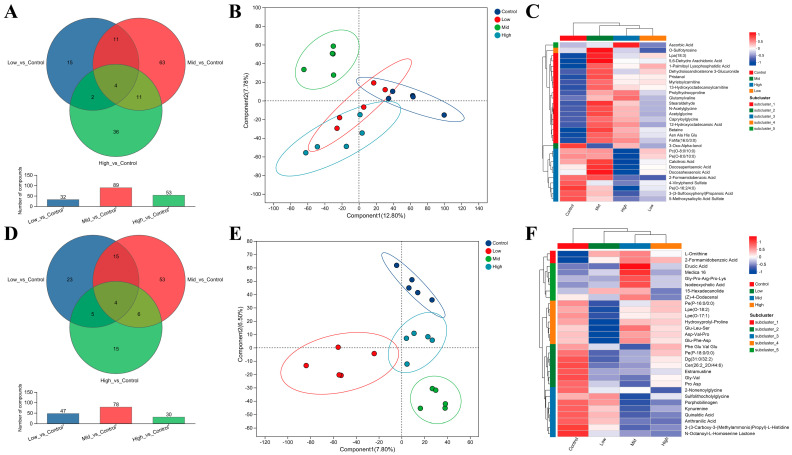
Serum differential metabolite profiling in ewes and lambs. (**A**) Venn diagram of serum differential metabolites among groups in ewes. (**B**) PLS-DA plot of multi-group comparison for serum differential metabolites in ewes. (**C**) Clustering heatmap of multi-group differential metabolites in ewes. (**D**) Venn diagram of serum differential metabolites among groups in lambs. (**E**) PLS-DA plot of multi-group comparison for serum differential metabolites in lambs. (**F**) Clustering heatmap of multi-group differential metabolites in lambs.

Venn analysis revealed distinct serum metabolic alterations in response to RP-Gln supplementation. In ewes, the Low-, Mid-, and High-dose groups exhibited 32, 89, and 53 differential metabolites, respectively, compared with the Control group, with 4–15 shared metabolites across dose groups (Figure 5A). PLS-DA score plots showed clear separation between the Mid group and other groups, with no overlap with the Control group (Figure 5B). A similar separation trend was observed in lambs (Figure 5E), where 47, 78, and 30 differential metabolites were identified in the respective dose groups relative to the Control, indicating that RP-Gln supplementation significantly altered serum metabolic phenotypes in both ewes and their offspring in a dose-dependent manner (Figure 5D). In ewes, 21 metabolites showed peak abundance in the Mid group. These were primarily represented by polyunsaturated fatty acids and derivatives—including docosahexaenoic acid, docosapentaenoic acid, and 13-hydroxyoctadecanoylcarnitine—along with the antioxidant ascorbic acid and several tyrosine derivatives (Figure 5C). In lambs, 18 metabolites were most abundant in the Mid group, encompassing lipid molecules such as 15-hexadecanolide, phosphatidylethanolamine, diacylglycerol, and ceramide (Figure 5F). Elevated levels of 2-(3-carboxy-3-(methylammonio)propyl)-L-histidine suggested enhanced histidine metabolism. Upregulated porphobilinogen and nucleotide derivatives indicated modulations in pyrimidine metabolism, while detection of peptides containing D-amino acid residues reflected active D-amino acid metabolic activity in lambs (Figure 5F).

### 3.8. Effects of RP-Gln Supplementation in Ewes During Late Gestation on Serum Metabolic Pathways in Ewes and Their Offspring

**Figure 6:**
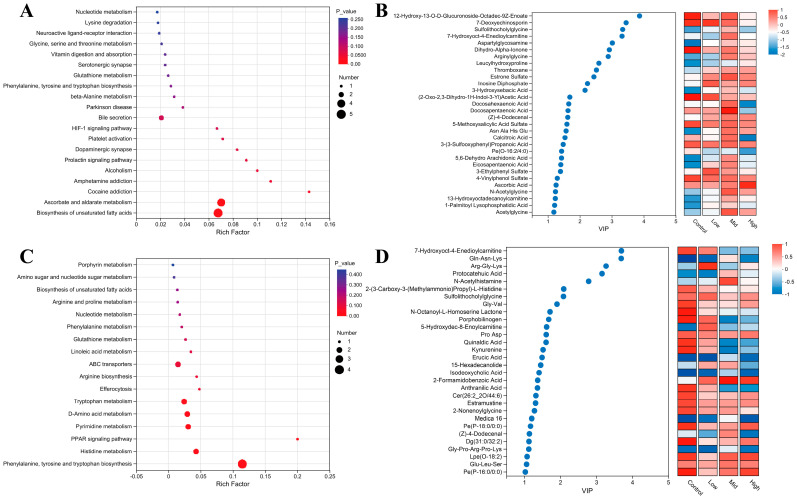
KEGG pathway enrichment analysis and VIP value analysis of serum differential metabolites in ewes and lambs. (**A**) Bubble plot of KEGG pathway enrichment analysis for serum differential metabolites in ewes. (**B**) Bubble plot of VIP values and heatmap of relative abundance for multi-group differential metabolites in ewes. (**C**) Bubble plot of KEGG pathway enrichment analysis for serum differential metabolites in lambs. (**D**) Bubble plot of VIP values and heatmap of relative abundance for multi-group differential metabolites in lambs.

KEGG pathway enrichment and variable importance in projection (VIP) analyses were performed to interpret the functional implications of the altered serum metabolomes. In ewes, 22 differential metabolites were identified, predominantly enriched in pathways related to lipid metabolism and antioxidant activity (Figure 6B). The biosynthesis of unsaturated fatty acids (map01040) was the most significantly enriched pathway (*p* < 0.05), followed by ascorbate and aldarate metabolism (map00053; *p* < 0.05; Figure 6A). VIP analysis highlighted an upregulation of polyunsaturated fatty acids (PUFAs)—including docosapentaenoic acid, docosahexaenoic acid, and 5,6-dehydroarachidonic acid—in the Mid group, whereas their levels declined in the High-dose group (Figure 6B). In addition, ascorbic acid content showed a dose-dependent increase with rising RP-Gln supplementation (Figure 6B). In lamb serum, 38 differential metabolites were detected, with significant enrichment in the phenylalanine, tyrosine, and tryptophan biosynthesis pathway (map00400; *p* < 0.05). Other pathways, including PPAR signaling, histidine metabolism, and pyrimidine metabolism, exhibited trends of modulation without reaching statistical significance (Figure 6C). VIP scores revealed markedly elevated levels of histidine metabolism-related metabolites (e.g., N-acetylhistamine) in Mid group lambs, whereas products of the kynurenine pathway—such as kynurenine and anthranilic acid—were significantly reduced (Figure 6D). Given the established roles of histidine in gluconeogenesis and enzyme activity regulation, together with the central position of the kynurenine pathway in tryptophan catabolism and immune modulation, these results suggest that moderate maternal RP-Gln supplementation promotes amino acid anabolism in offspring and attenuates the tryptophan–kynurenine degradation axis, which is often linked to pro-inflammatory responses.

### 3.9. Correlation Analysis of Rectal Microbiota-Metabolite Interactions and Their Associations with Oxidative and Inflammatory Status in Ewes and Their Offspring Supplemented with RP-Gln During Late Gestation

**Figure 7:**
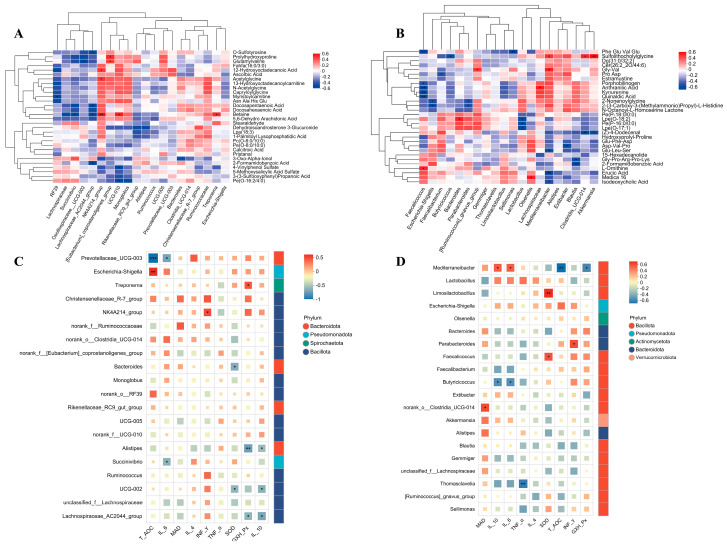
Correlation analysis between rectal fecal microbiota and serum antioxidant/inflammatory indicators and metabolites in ewes and lambs. (**A**) Heatmap of correlation analysis between genus-level rectal fecal microbiota and serum differential metabolites in ewes. (**B**) Heatmap of correlation analysis between genus-level rectal fecal microbiota and serum differential metabolites in lambs. (**C**) Heatmap of correlation analysis between genus-level rectal fecal microbiota and serum antioxidant parameters and inflammatory cytokine levels in ewes. (**D**) Heatmap of correlation analysis between genus-level rectal fecal microbiota and serum antioxidant parameters and inflammatory cytokine levels in lambs. * *p* < 0.05, ** *p* < 0.01, *** *p* < 0.001.

In ewes, correlation analysis revealed that only a limited number of abundant bacterial genera showed significant associations with antioxidant parameters and inflammatory cytokines (Figure 7A,C). *Prevotellaceae UCG-003* exhibited strong negative correlations with T-AOC and IL-6 (Figure 7C). *Succinivibrio* was significantly negatively correlated with IL-6, while the *NK4A214* group showed a positive correlation with IFN-γ (Figure 7C). *Bacteroides* and *Oscillospiraceae UCG-002* were both negatively correlated with SOD activity (Figure 7C). Both *Alistipes* and *Lachnospiraceae AC2044* group demonstrated negative correlations with GSH-Px activity and IL-10 levels (Figure 7C). Further analysis identified betaine—a microbially derived methyl donor—as a central metabolite mediating microbe-host metabolic interaction. It correlated positively with *NK4A214* group and *Oscillospirale UCG-010*, but negatively with *Lachnospiraceae AC2044* group and *Succinivibrio* (Figure 7A). *NK4A214* group also displayed positive correlations with acetylglycine and 12-hydroxyoctadecanoic acid (Figure 7A). *[Eubacterium]coprostanoligenes* group correlated positively with prolyl-hydroxyproline and glutamylvaline, whereas *Oscillospiraceae UCG-002* correlated negatively with the latter (Figure 7A). *Lachnospiraceae AC2044* group was negatively correlated with docosapentaenoic acid, 2-formamidobenzoic acid, and O-sulfo-L-tyrosine (Figure 7A). RF39 correlated negatively with lauroylcarnitine (Figure 7A). Although *Oscillospirale UCG-010* correlated positively with betaine, it showed negative correlations with PE(O-16:2/4:0) and 4-vinylphenol sulfate (Figure 7A). In lambs, *Clostridia UCG-014* was positively correlated with MDA levels (Figure 7B). Mediterraneibacter exhibited positive correlations with IL-10 and IL-6, but negative correlations with T-AOC and GSH-Px activity (Figure 7D). *Butyricicoccus* was negatively correlated with IL-10 and IL-6, while *Thomasclavelia* was negatively correlated with TNF-α. *Faecalicoccus* and *Limosilactobacillus* were positively correlated with SOD activity, and *Parabacteroides* was positively correlated with IFN-γ (Figure 7D). Correlation analysis between lamb fecal microbiota and serum metabolites indicated that *Faecalicoccus* was negatively correlated with multiple tryptophan metabolites—including 2-aminobenzoic acid, kynurenine, cholestanetrion, and quinolinic acid—as well as with the peptide Phe-Glu-Val-Glu, but positively correlated with L-ornithine (Figure 7B). *Parabacteroides* was negatively correlated with 2-[3-carboxy-3-(methylammonio) propyl]-L-histidine and 2-nonenoylglycine (Figure 7B). *Faecalibacterium* was negatively correlated with proline-aspartate, and *Butyricicoccus* was negatively correlated with glycolithocholate sulfate (Figure 7B). *Lactobacillus* was negatively correlated with several ether lipids, whereas *Bacteroides* was positively correlated with LPE (18:2) (Figure 7B). *Mediterraneibacter* showed positive correlations with Gly-Val and glycolithocholate sulfate (Figure 7B). *Clostridia UCG-014* and *Akkermansia* were both positively correlated with glycolithocholate sulfate and negatively correlated with (Z)-4-dodecenal; *Butyricicoccus* displayed the opposite correlation pattern (Figure 7B). *Sellimonas*, *Blautia*, and *Extibacter* were consistently negatively correlated with various peptides and aromatic metabolites (Figure 7B).

## 4. Discussion

The results of this study partially confirmed the initial hypothesis, demonstrating that supplementing ewes with an appropriate dose of RP-Gln during late gestation significantly improved lamb birth weight and promoted subsequent growth by enhancing antioxidant and anti-inflammatory capacities in both dams and offspring, enriching beneficial microorganisms, and reconstructing key metabolic pathways. However, its impact on maternal metabolism was primarily focused on lipid metabolism, with the medium-dose regimen proving optimal, while the high-dose regimen may induce adverse effects. These findings collectively suggest that RP-Gln supplementation may play an important potential role in regulating gestational health. Given that rumen-related parameters were not directly measured in this study, the following discussion will focus primarily on the potential effects of RP-Gln on intestinal function and examine its possible association with the observed changes in systemic indicators.

### 4.1. Mid-Level RP-Gln Supplementation as the Optimal Dose for Enhancing Growth Performance

First, the Mid-dose RP-Gln regimen produced the most pronounced improvement in lamb growth performance and therefore should be considered the optimal regimen among the doses evaluated, providing a strong entry point for deeper mechanistic exploration. In this trial, lambs in the Mid group exhibited significantly greater birth weight, day-15 body weight, and average daily gain (ADG) than those in both the Control and Low groups. This pattern is consistent with observations in monogastric models, where supplementing sows with 1% glutamine during late gestation increased piglet birth weight [26]. By contrast, High-dose supplementation failed to provide additional benefits and even exerted adverse effects on certain parameters. A plausible explanation is that excessive glutamine suppresses the nitric oxide synthase (NOS) pathway [27], thereby reducing nitric oxide (NO) production, diminishing placental perfusion, impairing transplacental nutrient transport, and ultimately restricting fetal growth [28]. Taken together, these findings reconcile the discrepancy between our initial expectation that the High dose would be most effective and the present data by indicating that RP-Gln exerts a threshold-dependent effect, with Mid-dose supplementation being optimal under the conditions of this study, whereas higher doses may override potential benefits and predispose animals to metabolic and vascular disturbances.

### 4.2. Improvements in Maternal and Offspring Metabolic and Antioxidant Status Following RP-Gln Supplementation

Beyond growth performance, RP-Gln also optimized metabolic and antioxidant defenses in ewes and their offspring by modulating key pathways. In ewes, RP-Gln significantly increased activities of γ-glutamyl transferase (GGT) and glutathione peroxidase (GSH-Px). By strengthening antioxidant defenses, glutamine likely confers metabolic protection and supports hepatic function, consistent with findings in rats [29]. In line with work in piglets showing that 1 g/kg body weight of supplemental glutamine during the suckling period promotes lipid deposition and muscle development in low-birth-weight piglets [30], our results indicate that appropriate RP-Gln provision to ewes in late gestation yields comparable benefits. Lambs from RP-Gln–supplemented ewes displayed higher total cholesterol, low-density lipoprotein, and high-density lipoprotein concentrations, together with reduced creatine kinase activity—patterns suggestive of improved lipid utilization and alleviated muscle stress. Mechanistically, glutamine-derived α-ketoglutarate can be converted to citrate, thereby fueling de novo lipogenesis [31]. Several poultry studies have reported dose-dependent enhancements in antioxidant capacity with glutamine supplementation [32,33,34]. Consistent with these reports, we observed increased GSH-Px activity in ewes and elevated superoxide dismutase (SOD) activity in lambs following appropriate RP-Gln supplementation, indicating a systemic upregulation of redox defenses. Biochemically, glutamine’s conversion to glutamate supports glutathione synthesis, which reduces dehydroascorbate back to ascorbate (vitamin C) [35]; accumulating ascorbate can further potentiate antioxidant enzyme activities, contributing to reduced oxidative damage [36]. Metabolomic profiling corroborated these physiological effects: Mid-dose ewes showed accumulation of polyunsaturated fatty acids, vitamin C, and tyrosine derivatives, while their lambs exhibited enrichment of lipids, dipeptides, and aromatic amino acids. These patterns echo observations from studies supplementing N-carbamylglutamate to nutrient-restricted ewes, which reported improved antioxidant capacity, amino-acid transport, and endocrine status associated with enhanced fetal growth [37]. Collectively, the evidence suggests that an appropriate dose of RP-Gln during late gestation optimizes hepatic metabolism and fortifies antioxidant defenses in the dam–offspring pair, thereby supporting neonatal adaptation to extrauterine life.

### 4.3. Dose-Dependent Immunomodulatory Effects and Gut Microbiota Remodeling Induced by RP-Gln

Beyond growth outcomes, a central action of RP-Gln is its coordinated regulation of immune status and gut microbiota. In poultry, glutamine suppresses LPS-induced hepatic expression of IL-1, IL-6, and TNF-α, thereby attenuating inflammation and promoting protein synthesis [38]. Mechanistically, glutamine can inhibit NF-κB activation by promoting deneddylation of Cullin-1, which downregulates IL-6 and TNF-α expression [39]. Our data align with this framework: IL-6 and TNF-α were reduced in High-group ewes, whereas IL-10 increased in the Mid group. Moreover, lambs in the High group exhibited higher IL-4 and IFN-γ concentrations, indicating a shift toward a more balanced Th1/Th2 milieu [40]. RP-Gln also elicited pronounced, dose-dependent remodeling of the gastrointestinal microbiota in both ewes and lambs, offering experimental support for vertical microbial transmission and early-life metabolic programming. Glutamine is known to shape microbial communities via multiple pathways, including activation of NF-κB and PI3K–Akt signaling, suppression of bacterial overgrowth and translocation, and enhancement in secretory IgA and IgA^+^ cell numbers [41].

### 4.4. Divergent Microbial Responses: Enrichment of Beneficial Taxa at Mid Dose Versus Pathogen Expansion at High Dose

Consistent with these effects, the Mid group showed significant enrichment of beneficial taxa—*Lactobacillus*, *Limosilactobacillus*, *Faecalibacterium*, and members of *Christensenellaceae*. These organisms are characterized by lactate and butyrate production, which supports intestinal development and exerts anti-inflammatory actions [42,43,44,45]. For example, *Faecalibacterium* possesses high butyrogenic capacity and, through interspecies cross-feeding, converts microbially derived acetate into butyrate, a key energy substrate for intestinal epithelial cells with well-described anti-inflammatory effects [46]. Butyrate-producing consortia also metabolize indigestible polysaccharides to lactate and other short-chain fatty acids (SCFAs), selectively fostering beneficial genera such as *Lactobacillus* while restricting pathogens via competitive exclusion and antimicrobial metabolites [47]. *Christensenellaceae*, which participate in cellulose and starch degradation, are positively associated with SCFA output [48]. In line with these functions, correlation analyses revealed positive associations between *Limosilactobacillus* and *Faecalibacterium* and circulating ascorbate, tyrosine derivatives, and PUFA levels, suggesting synergistic contributions to lipid metabolism and augmentation of antioxidant status.

By contrast, High-dose RP-Gln was accompanied by increased relative abundance of potential pathogens, including *Helicobacter* and *Tyzzerella*. Certain *Helicobacter* species are closely linked to gastrointestinal disease (e.g., peptic ulcers) [49], and *Tyzzerella*—an opportunistic pathogen—tends to be more active in spring [50], coinciding with the lambing season in our study. These adverse shifts may reflect ammonia accumulation and altered amino-acid transport under excessive glutamine intake. Long-term glutamine provision has been reported to perturb amino-acid profiles, increase ammonia production, and disrupt immune homeostasis [51].

The parallel hindgut microbiota patterns observed in ewes and lambs support the concept of maternal–offspring microbial transfer. Recent work indicates that the ewe’s gut microbiota can directly influence offspring colonization trajectories and growth performance [52], and early microbial assembly is a determinant of lamb birth weight and average daily gain [53]. Notably, enrichment of *Christensenellaceae*—a heritable microbiome member [54]—suggests that maternal nutritional modulation can shape inheritable hindgut microbial features.

Taken together, our findings indicate that appropriately dosed RP-Gln promotes hindgut colonization by commensal fermentative bacteria, suppresses opportunistic pathogens in the hindgut, and, through hindgut microbiota–mediated mechanisms, contributes to improved nutrient absorption and metabolic health in lambs.

### 4.5. Metabolomic Evidence for Maternal–Fetal Metabolic Reprogramming Under RP-Gln Supplementation

Metabolomic analysis offered deeper mechanistic insight into glutamine’s regulatory effects. In ewes, Mid-dose RP-Gln activated pathways linked to unsaturated fatty-acid biosynthesis and antioxidant defense, as evidenced by markedly elevated docosahexaenoic acid (DHA), eicosapentaenoic acid (EPA), ascorbate, and tyrosine derivatives. Contemporary studies indicate that glutamine supplementation can enhance polyunsaturated fatty-acid (PUFA) synthase activity and optimize lipid metabolism by lowering the n–6/n–3 PUFA ratio, thereby supporting overall health [55]. Consistent with this, José A. Roque-Jiménez et al. showed that supplementing pregnant ewes with DHA and EPA alters offspring fatty-acid composition in liver, muscle, and adipose tissues and modulates hepatic mRNA expression [56].

Together with evidence that maternal fermentation-derived short-chain fatty acids (SCFAs) can be vertically transmitted to promote immune maturation in offspring [44], these findings support a model in which RP-Gln during gestation augments in vivo synthesis of long-chain n-3 PUFAs (e.g., EPA and DHA) facilitates their placental transfer to the fetus [56]. This process may program lipid accretion during fetal development [57].

In lambs, upregulation of aromatic-amino-acid biosynthesis coincided with reduced kynurenine and quinolinic acid. Because quinolinic acid is an excitotoxic N-methyl-D-aspartate (NMDA) receptor agonist, its diminution may mitigate oxidative stress and bioenergetic dysfunction [58]. This metabolic reprogramming implies a redistribution of tryptophan flux away from the kynurenine pathway and toward serotonin biosynthesis [59], a circuit integral to systemic energy homeostasis [60]. Correlation analyses further revealed negative associations between kynurenine/quinolinic acid and birth weight, average daily gain, and heart, liver, and kidney weights, indicating that suppressing the kynurenine arm contributes to improved growth and organ development.

Collectively, these metabolomic signatures suggest that maternal glutamine supplementation exerts durable regulatory effects on offspring metabolic pathways, potentially via epigenetic mechanisms or endocrine signaling.

### 4.6. Limitations and Future Research Directions

Several limitations should be acknowledged. First, the placenta, uterus, and mammary gland were not directly examined; consequently, the cellular and tissue-level bases of nutrient transport and immunoregulation remain unresolved. Second, our metabolomic profiling was untargeted and therefore hypothesis-generating; targeted quantification of key metabolites is needed to validate the implicated pathways. Third, potential synergistic effects between glutamine and other functional amino acids or probiotics warrant investigation, as prior work has reported benefits from combining methionine with n-3 long-chain unsaturated fatty acids [61]. Fourth, it must be noted that a key limitation of this study is the absence of direct assessment regarding the effects of rumen-protected glutamine (RP-Gln) on ruminal fermentation parameters and microbial composition. Although a rumen-protection strategy was employed to reduce microbial degradation of glutamine in the rumen and thereby ensure its sufficient delivery to and absorption in the intestines, the lack of rumen sample data precludes clarification of the potential subtle disturbances this intervention may have exerted on the ruminal microbial network. Consequently, the physiological effects observed in this study should be interpreted cautiously as being primarily intestinal-mediated or systemic in nature, and the potential indirect contributions attributable to ruminal microbial metabolism cannot be distinguished or quantified here. Finally, it should be noted that the relatively small sample size in each group of this study, particularly for lambs subjected to histological and microbiota analyses, may limit the generalizability of the findings. This limitation should be taken into consideration when interpreting the conclusions.

## 5. Conclusions

This study suggests that glutamine supplementation during late gestation likely contributed to benefits for both the dam and offspring through multi-level mechanisms. At the maternal level, supplementation enhanced antioxidant defenses, dampened inflammatory responses, optimized hindgut microbiota composition, and promoted synthesis of n-3 PUFAs, notably EPA and DHA. At the fetal/lamb level, improved maternal antioxidant capacity and lipid mediators were transferred across the placenta, thereby elevating lamb antioxidant status, immune function, and intestinal health. Concomitantly, supplementation enriched beneficial microbial colonization, upregulated aromatic-amino-acid biosynthesis, and suppressed tryptophan degradation via the kynurenine pathway, reducing the accumulation of excitotoxic metabolites. Collectively, these coordinated effects contributed to improved lamb growth performance and organ development. These findings provide a mechanistic basis for deploying functional amino acids in ruminant production and offer a practical nutritional strategy to enhance the efficiency of sheep systems.

## Figures and Tables

**Table 1:** Composition and nutritional level of total mixed diet (dry matter basis).

Ingredient, % of DM ^1^	Content
Corn silage	20.00
Alfalfa hay	25.00
Chinese wildrye hay	20.00
Corn	19.00
Soybean meal	8.00
Corn bran	6.00
Monocalcium phosphate	0.50
Salt	0.50
Premix ^2^	1.00
Nutritional, % of DM ^1^	
Metabolic energy ^3^, MJ/kg	9.04
Crude protein, %	13.51
Ether extract, %	2.96
Neutral detergent fiber, %	43.45
Acid detergent fiber, %	27.21
Crude ash, %	0.59
Calcium, %	0.73
Phosphorus, %	0.44
Rumen degradable protein, %	8.99
Rumen undegraded protein, %	4.52
Lysine, %	0.64
Methionine, %	0.21
Threonine, %	0.49

^1^ DM, dry matter, dry matter basis. ^2^ Each kilogram of premix contains: vitamin A 6000 KIU, vitamin D3 210 KIU, vitamin E 4500 IU, iron 7000 mg, copper 1400 mg, zinc 6000 mg, manganese 5000 mg, selenium 60 mg. ^3^ Metabolic energy is a calculated value, referenced from *Nutrition Requirements of Meat-type Sheep and Goat (NY/T 816-2021)*; all other values are measured.

**Table 2:** Effects of Adding Different Levels of Rumen-Protected Glutamine on Colostrum and Regular Milk Quality.

Items	Groups				SEM	*p*-Value
	Control	Low	Mid	High		
Colostrum (%)						
Milk fat	11.66 ^a^	9.29 ^b^	12.06 ^a^	10.86 ^ab^	0.54	0.01
Non-fat milk solids	21.74	19.99	21.02	20.20	1.53	0.84
Lactose	9.76	8.97	8.76	9.07	0.80	0.83
Solids	1.66	1.52	1.61	1.54	0.11	0.80
Milk protein	10.30	9.48	9.95	9.56	0.72	0.84
Regular milk (%)						
Milk fat	4.15	4.72	4.44	2.85	0.54	0.13
Non-fat milk solids	9.38	9.03	9.09	10.56	1.53	0.12
Lactose	4.20	4.05	4.08	4.74	0.80	0.12
Solids	0.71	0.69	0.69	0.79	0.11	0.17
Milk protein	4.44	4.27	4.30	5.00	0.72	0.12

^ab^ If there is no same letter in the same data, it means that it is significant, *p* < 0.05.

**Table 3:** Effects of Adding Different Levels of Rumen-Protected Glutamine on Serum Biochemical Parameters in Ewes.

Items	Groups				SEM	*p*-Value
	Control	Low	Mid	High		
Ewes						
ALT (U/L)	16.70	16.80	15.90	17.40	2.27	0.95
TP (g/L)	65.39	66.94	59.96	64.27	2.62	0.11
ALB (g/L)	22.86	24.64	22.14	23.53	1.01	0.16
GLB (g/L)	42.53	42.39	37.82	40.74	2.23	0.20
A/G	0.55	0.60	0.60	0.60	0.04	0.54
AST (U/L)	85.00	83.60	68.60	78.00	6.00	0.07
GGT (U/L)	57.30 ^b^	65.20 ^ab^	70.70 ^a^	62.40 ^ab^	4.26	0.05
TG (mmol/L)	0.23	0.25	0.19	0.20	0.04	0.62
TC (mmol/L)	1.73	1.91	1.75	1.63	0.14	0.34
LDL (mmol/L)	0.50	0.52	0.55	0.44	0.07	0.49
HDL (mmol/L)	0.95	1.04	0.89	0.94	0.06	0.19
CK (U/L)	104.43	134.23	132.60	146.90	24.75	0.46
HBDH (U/L)	376.9	383.40	363.63	360.61	32.50	0.91
LDH (U/L)	382.06	365.67	387.82	365.49	33.84	0.90
Lambs						
ALT (U/L)	7.00 ^ab^	9.40 ^a^	6.20 ^b^	6.00 ^b^	0.76	0.02
TP/(g/L)	64.30	66.12	66.68	65.34	1.39	0.65
ALB (g/L)	22.56	22.96	23.02	22.68	0.59	0.93
GLB (g/L)	41.74	43.16	43.66	42.86	1.28	0.75
A/G	0.56	0.54	0.56	0.54	0.03	0.95
AST (U/L)	42.00	41.60	41.20	48.80	2.17	0.08
GGT (U/L)	124.20 ^b^	122.60 ^b^	216.60 ^a^	152.60 ^b^	15.04	<0.01
TG (mmol/L)	0.48	0.57	0.55	0.53	0.04	0.41
TC (mmol/L)	1.94 ^b^	2.42 ^ab^	2.84 ^a^	3.03 ^a^	0.20	0.01
LDL (mmol/L)	0.54 ^b^	0.72 ^ab^	0.93 ^a^	0.98 ^a^	0.07	<0.01
HDL (mmol/L)	1.03 ^b^	1.19 ^ab^	1.33 ^ab^	1.43 ^a^	0.08	0.01
CK (U/L)	356.84 ^a^	289.50 ^a^	211.76 ^b^	225.38 ^b^	17.96	<0.001
HBDH (U/L)	409.12 ^b^	494.24 ^a^	401.76 ^b^	468.70 ^ab^	19.39	0.01
LDH (U/L)	494.88 ^ab^	566.42 ^ab^	484.08 ^b^	579.58 ^a^	21.80	0.01

^ab^ If there is no same letter in the same data, it means that it is significant, *p* < 0.05. Abbreviations are defined in the Abbreviation section.

## Data Availability

All raw sequencing data have been deposited in the NCBI Sequence Read Archive under BioProject accession number PRJNA1294606 (https://www.ncbi.nlm.nih.gov/bioproject/PRJNA1294606, accessed on 23 July 2025).

## Abbreviations

The following abbreviations are used in this manuscript:

A/G	Albumin-to-Globulin Ratio
ALB	Albumin
ALT	Alanine Aminotransferase
AST	Aspartate Aminotransferase
CK	Creatine Kinase
GGT	Gamma-Glutamyl Transferase
GLB	Globulin
GSH-Px	Glutathione Peroxidase
HBDH	Hydroxybutyrate Dehydrogenase
HDL	High-Density Lipoprotein
IFN-γ	Interferon-gamma
IL-10	Interleukin-10
IL-4	Interleukin-4
IL-6	Interleukin-6
LDH	Lactate Dehydrogenase
LDL	Low-Density Lipoprotein
MDA	Malondialdehyde
PUFAs	Polyunsaturated fatty acids
RP-Gln	Rumen-protected glutamine
SOD	Superoxide Dismutase
T-AOC	Total Antioxidant Capacity
TC	Total Cholesterol
TG	Triglycerides
TNF-α	Tumor Necrosis Factor-alpha
TP	Total Protein
